# Biological agents targeting beyond TNF-alpha

**DOI:** 10.4103/0972-5229.45079

**Published:** 2008

**Authors:** Rashmi Sharma, Chaman Lal Sharma, Annil Mahajan

**Affiliations:** **From:** Department of Pharmacology and Therapeutics, Govt. Medical College, Jammu (J and K) -180 001, India; 1 Department of Pharmacology and Therapeutics, Indian-III Hospital, UN Mission Congo (Monuc), Armed Medical Corps, India; 2 Department of General Medicine, Govt. Medical College, Jammu (J and K), India

**Keywords:** Biological agents, cytokines, CD20 antigen, rheumatology, T-cells

## Abstract

Biological agents represent an important addition to the therapies for immuno-inflammatory conditions and have a great impact on the disease course and quality of life of these patients. However, recent reports of serious infections like tuberculosis, demyelinating and neurodegenerative diseases, pancytopenia, cardiovascular diseases, etc. after anti-TNF therapy raised questions on their safety. Hence, focus is shifted towards drugs targeting cytokine checkpoints in the inflammatory cascades beyond TNF-α. Existing therapeutic targets include the biological agents acting as antagonists of various inflammatory cytokines (Anakinra, Tocilizumab, Atlizumab) and modulators of CD80 or CD86-CD28 co-stimulatory signal (Abatacept), CD2 receptors on T-cells (Alefacept), CD11a, subunit of leukocyte function-associated antigen 1 (Efalizumab), vitronectin receptor and CD20 antigen on pre-B, immature and mature B cells (Rituximab). With the introduction of these novel molecules the future for immunomodulatory intervention in rheumatology, asthma, crohn's disease, septic shock etc. looks very promising. These novel therapeutic agents could truly give a new hope to the clinician to modify the disease and achieve tangible improvements in the lives of the patients.

Biologicals are proteins produced by living organisms to target specific sites of the inflammatory cascade, including antibodies against cell surface markers, cytokines and adhesion molecules.[1] The biological agents represent an important addition to the therapies for immuno-inflammatory conditions and have a great impact on the disease course and quality of life of these patients. However, recent reports of reactivation of TB (tuberculosis) after anti-TNF therapy raised question on their safety. TNF-α plays an important role in the host defense against mycobacterial infection, particularly in granuloma formation and inhibition of mycobacterial dissemination.[2] FDA recommended a black box for TB on the product labeling of infliximab.[4] Other serious infections reported with etanercept include sepsis secondary to Listeria monocytogenes and Histoplasma capsulatum.[2] Severe disseminated opportunistic infections have been reported in the HIV positive patients.[2] SLE syndrome, demyelinating diseases, neurodegenerative diseases, pancytopenia, cardiovascular diseases, new onset or flare-up of chronic iridocyclitis, thyroid cancer, hypoglossal nerve paralysis, severe cytomegalovirus pulmonary infection, reactivation of Crohn's disease etc. are the other important adverse effects reported with anti-TNF-α therapy.[4,5]

However, the success of TNF blockade clearly indicates that there are various checkpoints in cytokine-mediated inflammation. Hence, the identification and development of molecules targeting such critical ‘regulatory cytokines’ (beyond TNF) may have the potential to become a novel addition in the armamentarium against various immuno-inflammatory conditions. Hence, in the present article we are reviewing some of these non-TNF-α biological.

**Search Methodology:** Prominent rheumatology and general/internal medicine journals (MEDLINE, EMBASE, PUBMED between 2000 and 2006) were searched for review papers and clinical trials published on drugs targeting inflammatory cytokines other than TNF-α. All the data was collected and important evidences regarding pharmacology and uses of non-TNF-α biologicals were summarized in the present article.

## Cytokine networks and their therapeutic targets in clinical practice[6–8]

Cytokines are soluble (glyco) proteins, non-immunoglobulin in nature, released by living cells of the host, which act non-enzymatically in picomolar to nanomolar concentrations through specific receptors to regulate host cell function. Cytokines are pleiotropic in their biological activities and play pivotal roles in a variety of responses, including the immune response, hematopoiesis, neurogenesis, embryogenesis and oncogenesis. The main types of cytokines are lymphokines, interleukins, monokines, tumor necrosis factors (TNF), interferons, colony-stimulating factors, transforming growth factors, peptide growth factors, heat shock and other stress proteins. Cytokines have been classified on the basis of their biological responses into pro- or anti-inflammatory cytokines, depending on their effects on immunocytes [Table 1]. TNF, interleukin (IL)-1, IL-6, IL-7, IL-8, IL-12, IL-15, IL-17, IL-18, IL-23, lymphotoxin, macrophage migration inhibitory factor, resistin, interferon-γ, granulocyte-macrophage colony-stimulating factor, macrophage colony-stimulating factor, fibroblast growth factor and vascular endothelial growth factor are the proinflammatory cytokines. Whereas, IL-1Ra, IL-18 binding protein, IL-10, transforming growth factors, IL-11, IL-13, osteoprotegerin, adiponectin, etc are the anti-inflammatory cytokines. IL-22, oncostatin M etc are equivocal. Cytokines can be released not only by immune cells but also by host tissue cells. Various molecules (anakinra, tocilizumab, atlizumab, abatacept, alefacept, efalizumab, rituximab) targeting these cytokines are in clinical development.

**Table 1:** Cytokines and their role[6–8,43,44]

Cytokines	Function
IL-1	Activates APC and CD4+ lymphocytes; affects the differentiation of the B and T-Cells and other immunocompetent cells, takes part in the regulation of productions of other cytokines and GMCSF.
IL-2	Stimulates the proliferation and activation of B-Cells and T-Cells.
IL-4	Plays a role in the differentiation of TH2, in allergic responses and in the switching of antibody types.
IL-5	Stimulates the production and maturation of eosinophils during inflammation.
IL-8	Acts as a chemotactic factor that attracts neutrophils, basophils and T-Cells to sites of inflammation.
IL-12	A critical linker between the innate immunity and adaptive immunity, capable of TH1 differentiation and IFN-Gamma release by T-Cells and NK cells
IL-10	Acts to repress secretion of pro-inflammatory cytokines.
IL-3	Potent activator of the hemopoietic cells. It stimulates NK-Cells and acts as a synergist with IL-4 during the induction of CD4+ lymphocyte activation process.
IL-7	Induces apoptosis of tumor cells and causes differentiation of cells from a subgroup of acute myeloblastic leukemia.
IL-9	Stimulates the excretion of IL-2, IL-4, IL-6, IL-11 and cytotoxicity of T-killers and NK-Cells, inducing apoptosis.
IL-11	Regulates the functions of B-Cells and T-Cells, induces various killer cells' activities and acts as an autocrine factor for the proliferation of megacaryocytes.
IL-13	Inhibits the proliferation of leukemic pro-B-Cells
IL-14	BCGF and the hyper production of this interleukin enables the progression of NHL-B.
IL-15	Increases the antitumor activities of T-killers and NK-Cells, and the production of cytokines CD4+ lymphocytes.
IL-17	Takes part in the regulation of many cytokines and can reinforce the antibody dependant tumor cell destructions.
IL-18	Synergist with IL-12, especially in the induction of IFN-Gamma production and inhibition of angiogenesis.
IL-19	Regulates the functions of macrophages and suppresses the activities of TH1 and TH2.
IL-21	Promotes a high production of T-Cells, fast growth and maturation of NK-Cells and B-Cells population.
IL-22	Similar to IL-10, but does not prohibit the production of pro-inflammatory cytokines through monocytes.
TNFs	Activates macrophages, inhibits apoptosis of neutrophils and eosinophils, induces vascular endothelial cells to bind to phagocytes, induce proliferation of NK-Cells and stimulate innate and adaptive immune responses.
	On activation NK cells release IFN-γ.
Resistin	Promote TNF and IL-6 release.
Adiponectin	It modulates TNF-induced inflammation.

APC= Antigen presenting cell, GMCSF =Granulocyte-Macrophage Colony-Stimulating Factor, TH2 =T Helper Type-2, TH1 =T Helper Type-1, NK= natural killer, INF= interferone, IL=interleukins, BCGF=B-Cell Growth Factor, NHL-B =B-cell type non Hodgkin's lymphoma.

Existing therapeutic targets include the biologicals acting as antagonists of various inflammatory cytokines and modulators of CD80 or CD86-CD28 co-stimulatory signal, CD2 receptors on T-cells, CD11a, sub-unit of leukocyte function-associated antigen 1 (LeFA-1), vitronectin receptor and CD20 antigen on pre-B, immature and mature B cells [Table 2].

**Table 2:** Biologics agents beyond anti-TNF-therapy [2–12,18–35]

Drugs	Binding target	Dose	Adverse drug reaction	FDA approval
Anakinra a recombinant human IL-1 {RA}	IL-1 receptor	1-2 mg/kg/day S/C.	Neutropenia, cardiopulmonary arrest, influenza like symptoms, production of anti anakinra antibodies and serious infections.	RA(2001), Studied in Asp, psoriasis and PA.
Atlizumab: Mab	IL-6 receptor	2-8 mg/kg I.V. every 2 wkly	Increased blood cholesterol levels.	Studied in RA.
Abatacept: a recombinant fusion protein	Selectively modulates the CD80 or CD86-CD28 co-stimulatory signal required for full T-cell activation	10 mg/kg I.V. every 2 wkly for 3 doses followed by 4 wkly.	--	RA(2005) Studied in Asp, psoriasis and PA, CD-UC.
Rituximab: specific mouse and human chimeric Mab	CD 20 antigen on B cells	----	Antibody levels against HSV 1/2 and VZV are not significantly affected	Cancers, B-cell NHL(2001), RA(march 2006). Studied in Asp, psoriasis, PA
Alefacept a bivalent recombinant fusion protein	LFA-3 portion of alefacept binds to CD2 receptors on T-cells, IgG1 portion of alefacept binds to Fc▭R receptor on natural killer cells to induce T-cell apoptosis.	10-15 mg IM wkly or 7.5 mg IV wkly for 12 wks.	Cytotoxic effect is selective for the activated memory T-cells. reduces total lymphocyte count and CD4+ and CD8+cell counts.	Psoriasis(jan 2003),
Efalizumab a recombinant humanized IgG1 Mab	It interferes with the interaction between LeFA-1 and ICAM-1, a cell surface molecule expressed by APCs.	1 mg/kg (max 200mg) wkly S.C. for 12wks	-Acute flu- like symptoms, exacerbation of psoriasis on discontinuation, autoimmune hemolytic anemia, thrombocytopenia	Psoriasis(2003)
Vitaxin humanized monoclonal IgG1 antibody	Antagonizes vitronectin receptors involved in osteoclast mediated bone resorption, angiogenesis and macrophage dependent inflammation.	---	---	Early stages of study in RA.

Asp= ankylosing spondylitis, RA= Rheumatoid arthritis, APCs= antigen presenting cells, LeFA-1 = leukocyte function-associated antigen 1, ICAM-1 = intercellular adhesion molecules, LFA-3 = lymphocyte function antigen 3, CTLA4 = cytotoxic T-lymphocyte antigen 4, IL = Interleukin, PA =psoriatic arthritis, CD-UC = crohn's disease and ulcerative colitis, Mab = monoclonal antibody, HSV=.herpes simplex virus, VZV= varicella-zoster virus,{RA}= receptor antagonist, wkly = weekly, NHL= non-Hodgkin's lymphoma, S.C=subcutaneous,Wks= weeks.

**Anakinra:**[9–11] It is recombinant form of nonglycosylated human IL-1 receptor antagonist expressed in Escherichia coli. Natural IL-1 receptor antagonist is produced by macrophages and activated monocytes in response to various inflammatory stimuli. Anakinra competitively binds to both type-I and type-II IL-1 receptors, at least partially blocking cellular responses mediated by IL-1-α and IL-1-β. It has a binding affinity similar to IL-1, but it lacks IL-1 agonist activity. Its daily dose is 100 mg/day subcutaneously (SC). Prior hypersensitivity to anakinra or *E-coli* derived proteins and active infection are the important contraindications with the use of anakinra. Common adverse reactions reported with it are headache, nausea, diarrhea, sinusitis, erythema, ecchymosis, pruritis at injection site, influenza like symptoms, production of anti-anakinra antibodies, neutropenia, cardiopulmonary arrest and serious infections. Live vaccines should not be administered concurrently with anakinra. It should be used with caution in patients with neutropenia, immuno-suppression, moderate to severe renal impairment, pregnancy or breastfeeding period, and concomitant use of TNF blocking agents.

**Clinical Trials:** In a dosage ranging multi-center placebo controlled trial patients of Rheumatic arthritis (RA) on 1-2 mg/kg/day of anakinra with MTX 15-25 mg/week achieved more ACR (American College of Rheumatology preliminary criteria for improvement) 20 response than MTX alone at 12 weeks.[12] In another study with 100 mg/day of anakinra in combination with MTX showed more efficacy in retarding radiographic progression than MTX alone.[13] In a two-year prospective, in part retrospective, cohort study drug survival was 78%, 54%, and 14% after 3, 6 and 24 months, respectively.[14] However, National institute of clinical excellence of the united kingdom recommended its use in patients who are not responding to anti-TNFα therapy alone or in patients with juvenile idiopathic arthritis.[12,15]

In a clinical trial on 419 patients with moderate-to-severe active RA, who were receiving MTX for six consecutive months, the ACR20 responses at week 12 in the 5 active treatment (0.04, 0.1, 0.4, 1.0, or 2.0 mg/kg of anakinra) plus MTX groups demonstrated a statistically significant (*P* = 0.001) dose-response relationship compared with the ACR20 response in the placebo plus MTX group.[16] In another trial 218 patients received subcutaneous injections of anakinra (30, 75, or 150 mg) once daily.[17] The ACR20 response was 51% at week 24 and 46% at week 48 and this effect was consistent across all dose groups. Anakinra was well-tolerated for 76 weeks.[17] Role of anakinra in chronic infantile neurological cutaneous and articular (CINCA) syndrome with a novel missense mutation in exon 4 of the CIAS1 gene (unresponsive to several treatments including prednisolone, immunosuppressants, DMARDs and TNF-blocker infliximab) has been documented.[18] Anakinra, has a positive impact on both function and quality of life of the patients with RA.[19] However, further clinical studies are needed to establish the additive benefits of the combination of TNF-α blockade plus IL-1 receptor antagonism in RA.

**Abatacept:**[20] It is a recombinant fusion protein comprising of the extra-cellular domain of human CTLA4 (cytotoxic T-lymphocyte antigen 4) and a fragment of the Fc domain of human IgG1, which has been modified to prevent complement fixation. It modulates the CD80 or CD86-CD28 co-stimulatory signal required for full T-cell activation. It is given in a dose of 10 mg/kg by IV infusion (three doses at the interval of two weeks, followed by infusion after every four weeks).

**Clinical Trials:** In a Phase IIb multi-center international study in RA patients with inadequate response to MTX, ACR 20 response was achieved in 60%, 41.9% and 35.3% patients with abatacept in a dose of 10 mg/kg, 2 mg/kg and placebo respectively after 6 months of the treatment as add on therapy to MTX.[12] In a randomized double blind phase-III trial on patients with active RA refractory to anti-TNF-α therapy, abatacept therapy for 6 months, in addition to at least one DMRDs (disease modifying antirheumatic drugs) produced ACR 20 response rate of 50.4% as compare to 19.5% in the placebo group (*P*< 0.001).[20] At six months, significantly more patients in the abatacept group than in the placebo group had a clinically meaningful improvement in physical function (47.3 percent *vs* 23.3 percent, *P*<0.001) with incidence of serious infections as 2.3% in both the groups.[20]

**Alefacept:**[2] Alefacept is approved by US food and drug administration (FDA) in January 2003 for treatment in adult patients with moderate to severe chronic plaque psoriasis, who are candidates for systemic therapy or phototherapy. It is a bivalent recombinant fusion protein composed of the first extra-cellular domain lymphocyte function antigen 3 (LFA-3), fused to the hinge CH2 domain and CH3 domain of human IgG1. The LFA-3 portion of alefacept binds to CD2 receptors on T-cells, thereby blocking their natural interaction with LFA-3. The IgG1 portion of alefacept binds to FcγR on natural killer cells to induce T-cell apoptosis. Its dose is 10-15 mg IM (intramuscular) weekly or 7.5 mg IV (intravenous) weekly and a 12 week course is recommended.

**Clinical Trials:** In a double-blind RCT (randomized clinical trial) two 12-week courses of once-weekly IV alefacept 7.5 mg and placebo were given and patients were followed for 12 weeks after each course.[21] Significantly more patients achieved greater reduction in the PASI (psoriasis area and severity index) than placebo both after first and second course of therapy.[21] In an international, double-blind, placebo-controlled, RCT, 507 patients with chronic plaque psoriasis, were randomized to receive either 10 mg or 15 mg of alefacept once weekly for 12 weeks, followed by 12 weeks of observation.[22] Thirty three percent and 28% patients achieved 75% reduction in PASI, two weeks after the last dose in 15 mg and 10 mg group respectively.[22] The selective immuno-modulatory effect of alefacept against potentially pathogenic T-cells is associated with maintenance of immune function to fight infection and response to vaccinations.[23] It has been reported that it reduces total lymphocyte count and CD4+ and CD8+cell counts. Hence, it is recommended to monitor CD4 counts weekly during therapy.

**Efalizumab:**[2,3] It is approved by the US FDA in October 2003 for the treatment of psoriasis. It is a recombinant humanized monoclonal IgG1 antibody that binds to CD11a, subunit of leukocyte function-associated antigen 1 (LeFA-1). It interferes with the interaction between LeFA-1 and intercellular adhesion molecules (ICAM-1). By destabilizing the binding of APCs (antigen presenting cells) and T-cells, it reduces the efficiency of initial T-cell activation in lymph nodes. It interferes with the secondary activation of memory-effector T-cells in the target tissues. Its dose is 1 mg/kg (max 200mg) weekly, subcutaneous injection for 12 weeks, following a first conditioning dose of 0.7 mg/kg.

**Clinical trials:** In four large phase III studies in 2000 patients with moderate-to-severe chronic plaque psoriasis, efalizumab (1 mg/kg weekly) produced PASI 75 (>75% in reduction in baseline PASI score) in 27% of patients as compared to 4% patients in placebo group by week 12.[24–27] Continuation of therapy beyond 12 weeks increased the response rate further in efalizumab group. The relapse of psoriasis was evident after 2 months of discontinuation of therapy with rebound in approximately 5% of the patients, as defined by flaring>125% of baseline.Acute flu-like symptoms including headache, chills, fever, nausea and myalgia, an exacerbation of psoriasis after discontinuation of therapy, autoimmune hemolytic anemia, and thrombocytopenia are the common adverse events reported with its use.[2]

**Rituximab:**[28–30] Rituximab is a specific mouse and human chimeric monoclonal antibody. This IgG1 has a long half-life of 76 to 200h and targets the CD20 antigen. The CD 20 antigen is present on pre-B, immature and mature B cells and is important for B-cell activation and proliferation. Binding of rituximab to CD 20 results in complement and antibody-dependent cyto-toxicity (apoptosis) of cells exhibiting this antigen. CD20 is not expressed on stem cells and plasma cells. Hence, depletion of the B-cell subpopulation is transient and does not affect immunoglobulin synthesis. Normal levels of total serum IgG are maintained and antibody levels against HSV (herpes simplex virus) 1/2 and VZV (varicella-zoster virus) are not significantly affected after rituximab treatment. Rituximab was the first therapeutic antibody approved for treating cancer. A supplemental Biological License Application (sBLA) was approved for it in April 2001, adding several new uses related to B-cell non-Hodgkin's lymphoma. In 2006 rituximab in combination with MTX is approved for adult patients with moderately-to-severely active RA, who have had an inadequate response to one or more TNF antagonist therapies.

**Clinical Studies:** REFLEX, a Phase III clinical study of Rituximab in RA, met its primary endpoint and underpins the FDA's approval.[29] It is also being evaluated in Phase II/III clinical trials for primary progressive and relapsed remitting multiple sclerosis, ANCA-associated vasculitis, systemic lupus erythematosus.[29] In an open label study Rituximab in combination with cyclophosphamide and prednisolone in five patients of refractory RA showed dramatic and sustained clinical improvement.[31] In a phase III multi-center double blind trial on 161 patients Rituximab in combination with MTX or cyclophosphamide showed more efficacy than MTX alone.[31] Rituximab is the first treatment for RA that selectively targets immune cells known as CD20-positive B-cells.

**Atlizumab:**[12,32–35] It is a humanized anti-IL-6 receptor monoclonal antibody. It is efficacious in management of RA in a dose of 2-8 mg\kg\dose IV once every two weeks. It is a well-tolerated drug without any increase in antinuclear, anti-DNA or anti-atlizumab antibody. However, increase in blood cholesterol levels has been reported after its use for 24 weeks.

**Clinical Trial:**[12] In a phase I/II double blind RTC, atlizumab (5 mg/kg with MTX single dose) produced ACR20 response in 50% patients of RA as compare to placebo at week two. Improvement was maintained for eight weeks. In a multi-center double blind RCT, 78%, 57% and 11% RA patients achieved ACR20 response after three months of therapy with atlizumab 8 mg/kg, 4 mg/kg and placebo respectively.

**Tocilizumab:**[36,37] It is a recombinant humanized anti-IL-6R monoclonal antibody. Phase I and II studies of tocilizumab in children with JIA (juvenile idiopathic arthritis), showed significant improvement in the typical symptoms of inflammation and laboratory abnormalities.

**AMG714 (previously HuMax-IL15):**[6] IL-15 enhances synovial T-cell proliferation and cytokine release and optimizes cognate interactions between T cells and macrophages. IL-15 induces synovial neutrophil activation, granule release from natural-killer cells, activation and migration of endothelial cells and prevents fibroblast apoptosis. AMG714, a fully human IgG1 monoclonal anti-IL-15 antibody, neutralizes soluble and membrane-bound IL-15 *in vitro*.

**Clinical Trials:** In a 12-week, dose-ascending, placebo-controlled study, AMG714 (0.5–8 mg/kg) produced significant improvement in disease activity in RA patients as compared to placebo.[38] In another dose-finding study 60% of recipients receiving higher doses of AMG714 (160 mg or 240 mg) showed significant improvement as compared to lower doses.[6] No significant alterations in the levels of circulating leukocyte subsets, including natural-killer cells and CD8^+^ memory T cells, were observed.

Alternate approaches to targeting IL-15 include the use of soluble IL-15R-α-derived proteins or antagonistic IL-15–Fc fusion proteins. CRB-15, an IL-15–Fc fusion protein, suppressed delayed-type hypersensitivity and allograft transplant rejection in rodent models.[39]

Vitaxin (MEDI-522):[4] It is humanized monoclonal IgG1 antibody that binds to a conformational epitope formed by both the integrin alpha V and beta 3 subunits. Alpha V and beta 3 integrin (vitronectin receptor) is expressed in low levels in most of the normal tissues (intestinal, vascular and smooth muscle cells) and in high levels in bone, mid-menstrual cycle endometrium, placenta, inflammatory sites and invasive tumors. Vitronectin receptors have major role in osteoclast mediated bone resorption, angiogenesis and macrophage dependent inflammation. In RA, activated macrophages are increased in both subchondrial bone and inflamed synovial tissue; whereas, osteoclasts are increased in subchondrial bone at the site of bone erosion and resorption. Hence, antagonists of alpha V and beta 3 integrin have a potential role in the therapeutics of RA. Echistatin is another molecule under development.

## Anti Interleukins in Critical Pathological Conditions

**Bronchial Asthma:**[40,41] Accumulation of eosinophils in the bronchial mucosa of individuals with asthma is considered to be a central event in the pathogenesis of asthma. Mepolizumab is a humanized anti–IL-5 monoclonal antibody. In animal models, airway eosinophil recruitment and airway hyperresponsiveness in response to allergen challenge are reduced by specific targeting of IL-5. However mepolizumab treatment does not appear to add significant clinical benefit in patients with asthma with persistent symptoms despite inhaled corticosteroid therapy.

In a clinically relevant model of chronic allergic asthma in mice neutralizing antibodies to IL-13 effectively suppressed eosinophil recruitment and accumulation of chronic inflammatory cells in the airways. It also partially suppressed changes of airway wall remodeling, including goblet cell hyperplasia/metaplasia and subepithelial fibrosis, but had limited ability to inhibit airway hyperreactivity (AHR). However, treatment with anti–IFN-γ markedly suppressed AHR.

**Crohn's disease:**[43] Crohn's disease is characterized by increased production of IL-12 by antigen-presenting cells in intestinal tissue and interferon-γ and TNF-α by intestinal lymphocytes and macrophages. Anti–IL-12 monoclonal antibody therapy induces clinical response and remission in patients with active Crohn's disease. In a clinical trial 79 patients with active Crohn's disease were randomized to receive seven weekly subcutaneous injections of anti– IL-12 human monoclonal antibody (1 mg/kg body weight or 3 mg/kg body weight) or placebo, in an interrupted (one-month span after first dose, n = 40) or continuous regimen (n = 39). Continuous weekly therapy with 3-mg/kg anti–interleukin-12 resulted in significantly higher response rates at seven weeks compared with placebo.

**Acute respiratory distress syndrome:**[42] Anti-IL8 antibodies are high in patients of acute respiratory distress syndrome and molecules targeting them can be of potential help in these patients.

Septic shock: [44] Patients with septic shock have T cell hyporesponsiveness and immune suppression, which, if persistent, are associated with increased mortality. In the murine cecal ligation and puncture (CLP) model of sepsis, it has been reported that early treatment with the anti-inflammatory cytokine IL-10 delays the onset of irreversible shock.

## Cytokine targets in preclinical development

Interferon (IFN)-γ blockade using a polyclonal anti-IFN-γ antibody has been shown to produce suppression of RA disease activity in a small RCT.[45] Suppression of IL-17, anti-IL-18 antibody and the inhibition of IL-18 secretion via inhibition of caspase 1or antagonism of the proinflammatory purinergic receptor P2X7 are other approaches under development. [6,46–49] Several adipokines (adeponectin) have been shown to modulates TNF-induced inflammation.[50,51] Resistin is a cysteine-rich secretory protein originally implicated in insulin resistance and atherogenesis. It is expressed at high levels in RA synovial tissues, can promote TNF and IL-6 release.[6,51] Therapeutic targeting of resistin offers the potential to modify not only local inflammation, but also the systemic insulin resistance that is characteristic of RA and other chronic inflammatory conditions.

A number of biological agents are being studied actively at the present time and it is hoped that they may generate novel therapies for and a greater understanding of immuno-inflammatory diseases (Table 3). The future for immunomodulatory intervention in rheumatology looks very promising. Greater understanding of the intricacies of the immune response that underlie the disease should continue to yield viable, specific targets for novel therapies. Advances in biopharmaceuticals should generate treatments that maximize efficacy while minimizing toxicity. These novel therapeutic agents could give new hopes to the clinician truly to modify the disease and achieve tangible improvements in the lives of the patients.

**Table 3:** Clinical evidences showing efficacy of non-TNF biologic in immuno-inflammatory conditions

Study	Disease	Drugs	Duration	Results
DRM RCT	RA	Anakinra+MTX *vs* placebo+MTX	12 wks	>ACR 20 response in anakinra group than placebo group.[12]
Cohort study	RA	Anakinra	2 yr prospectively	Significant response at 3 months Survival 14% after 2 yrs.[14]
RCT	RA	Anakinra+MTX *vs* placebo+MTX	24 wks	ACR response was dose dependent and >ACR 20 response in anakinra group than placebo group.[16]
MDBP group extension phase study.	RA	Anakinra *vs* placebo	48wks	ACR 50 and ACR 70 responses are more in anakinra group than placebo group.[17]
Case report	CINCA	Anakinra	-	Improved condition in patient refractory to other DMRDs and biologicals.[18]
Phase II b multi-center international study	RA with inadequate response to MTX	Abatacept *vs* placebo.	6 months	ACR 20 response was achieved in 60%, 41.9% and 35.3% patients with 10mg/kg, 2 mg/kg abatacept and placebo respectively.[12]
Phase-III double blind RCT	RA refractory to anti-TNF-α therapy	Abatacept + 1 DMRD	6 months	ACR 20 response rate of 50.4% in abatacept group as compare to 19.5% in the placebo group (P<0.001); ACR 50 and ACR 70 responses were also higher in the abatacept group.[20]
Double-blind RCT	Psoriasis	Alefacept *vs* placebo	12 wks treatment and 12 wks follow-up.	Greater reduction in the PASI was achieved by alefacept group than placebo group ( *p* <0.001).[21]
International, double-blind, placebo-controlled, RCT	Chronic plaque psoriasis	Alefacept 10 mg or 15 mg once wkly	12 wks treatment and 12 wks follow-up	In the 15 mg group, 33% patients achieved 75% reduction in PASI, 2 wks after the last dose and 28% patients achieved 75% reduction in PASI in the 10 mg group.[22]
Four large phase III studies in 2000 patients	Chronic plaque psoriasis	Efalizumab *vs* placebo	>12 wks	27% of patients in efalizumab group, achieved PASI 75 compared to 4% in placebo group by wk 12.[24–27]
Phase I/II double blind RTC	RA	Atlizumab+MTX Single dose *vs* placebo	8 wks	50% patients achieved ACR20 in atlizumab group at wk 2,but non in placebo group.[12]
MDBP RCT	RA	Atlizumab *vs* placebo	3 months	78%, 57% and 11% patients achieved ACR20 response in atlizumab 8mg/kg, 4 mg/kg and placebo group respectively.[12]
Phase III MDBP	RA	Rtuximab+MTX or CP *vs* MTX	---	More improvement in Rituximab group.[31]

RCT = randomized control trial, RA= rheumatoid arthritis, PASI= psoriasis area and severity index., ACR= American College of Rheumatology preliminary criteria for improvement, CINCA = chronic infantile neurological cutaneous and articular, MTX= methotrexate, CP =cyclophosphamide, TNF = tumour necrosis factor, DMRDs = disease modifying anti-rheumtic drugs, DRM = dose ranging multicenter, MDBP = multicenter double blind parallel, wks =weeks.
